# Factors associated with seeking and access to oral health services among Brazilian adolescents and young adults

**DOI:** 10.1590/1980-549720260032.supl.1

**Published:** 2026-08-28

**Authors:** Rênnis Oliveira da Silva, Carolina Thaiza Costa Pazos, Ana Beatriz Vasconcelos Lima Araújo, Nilcema Figueiredo, Gabriela da Silveira Gaspar, Paulo Sávio Angeiras de Goes

**Affiliations:** I Universidade Federal de Pernambuco, Graduate Program in Child and Adolescent Health – Recife (PE), Brazil.; II Universidade Federal de Pernambuco, Department of Social Medicine – Recife (PE), Brazil.; III Universidade Federal de Pernambuco, Department of Public Health, Centro Acadêmico de Vitória – Vitória de Santo Antão (PE), Brazil.; IV Universidade Federal de Pernambuco, Department of Clinical and Social Dentistry – Recife (PE), Brazil.

**Keywords:** Equity in access to health services, Oral health, Dental health surveys, Adolescent

## Abstract

**Objective:**

To investigate individual and contextual factors associated with seeking and access to oral health services among Brazilian adolescents and young adults.

**Methods:**

This cross-sectional study was based on data from the 2023 Brazilian National Oral Health Survey (SB Brasil 2023). The sample comprised 12-year-old adolescents and young adults aged 15 to 19 years (n=14,757). Four outcomes structured into two stages (seeking and access) were assessed, subdivided into general oral health services and public services specifically. Binary logistic regression models were fitted to estimate odds ratios (ORs), and a 5% significance level was adopted (p<0.05).

**Results:**

All associations were statistically significant (p<0.001). Black race/skin color and income below the poverty line reduced the odds of seeking general oral health services (OR=0.86 and OR=0.84, respectively). For general access, socioracial inequities were more pronounced, intensifying the barrier for Black individuals (OR=0.79). Conversely, in the context of public services, the most vulnerable groups had the highest odds of both seeking and effectively accessing services, particularly Black individuals (OR_seeking_=2.72; OR_access_=2.57) and those with low income (OR_seeking_=2.31; OR_access_=2.38).

**Conclusions:**

Seeking and access to oral health services are influenced by socioeconomic and racial inequities. The public system acts as a compensatory resource for vulnerable groups but remains insufficient to overcome access barriers.

## INTRODUCTION

Oral health is an essential component of general health and physical, mental, and social well-being. It encompasses the ability to speak, smile, chew, savor, touch, swallow, and express emotions with confidence, without pain or discomfort in the craniofacial complex^1^. Access to health services is a complex phenomenon that has been analyzed from various perspectives. Its understanding was systematized through the Andersen Behavioral Model^2^ and the Modified Andersen Model^3^, which are widely used to explain the determinants of health service utilization. This theoretical framework has also been applied to the analysis of access to oral health care, allowing for an understanding of how predisposing factors, enabling factors, and needs influence the use of dental services^4,5^.

Adolescence and youth constitute critical phases of the life course, marked by intense behavioral changes, progressive acquisition of autonomy, and consolidation of health habits. The way dental care is accessed and utilized during this period can have repercussions throughout adult life, as unaddressed needs tend to worsen over time and exacerbate inequalities in oral health^6,7^.

In Brazil, oral health care is guaranteed by the Unified Health System (Sistema Único da Saúde – SUS) through the National Oral Health Policy, instituted in 2004 and established by Law No. 14.572 of May 8, 2023, known as the Sorridente Brazil Policy^8,9^. Despite this, access to oral health services remains marked by significant inequalities, particularly among low-income and less-educated groups and residents of vulnerable territories^6,8^.

Considering the persistence of inequalities and the scarcity of research that simultaneously evaluates access-seeking behavior from the perspective of individual and contextual determinants, this study is based on the hypothesis that socioeconomic and racial vulnerabilities reduce the likelihood of service utilization, while structural factors, such as healthcare coverage and school enrollment, may act as important mediators of equity. In this context, the pursuit of oral health care organizational models based on components that promote equity is imperative for oral health research in Latin America^10^.

The objective of this study was to investigate the individual and contextual factors associated with seeking and accessing oral health services among Brazilian adolescents and young people.

## METHODS

### Study design

This was an observational, cross-sectional, descriptive, and analytical study based on microdata from the Brazilian National Oral Health Survey (*Pesquisa Nacional de Saúde Bucal* – SB Brasil) 2023^11^. Detailed information on the methodological aspects and sample development of the study is available in publications by Vargas et al.^12^ and Alves et al^13^.

The SB Brasil 2023 study follows the ethical principles established in Resolution No. 466/2012 of the National Health Council and was approved by the National Research Ethics Committee (Resolution No. 4.823.054). Participation was voluntary and was granted through the Informed Consent and the Assent Forms, when applicable.

### Sample

The study sample comprised adolescents aged 12 years and young people aged 15 to 19 years who responded to the individual questionnaire of the SB Brasil 2023 survey (n=14,757). The survey sample was selected using a probabilistic, cluster-stratified sampling design (census tracts), ensuring population representativeness^12,13^. The SB Brasil 2023 sampling framework comprises 53 geographic domains, corresponding to the 26 states, the Federal District, and the state capitals^13^.

### Data collection

Individual-level data (seeking and accessing oral health services, dental caries experience, per capita household income, and self-reported race/ethnicity) and contextual data (type of municipality) were obtained from the National Oral Health Survey database^14^.

Additional contextual information on oral health coverage in Primary Care was obtained from the Health Information System for Primary Care (*Sistema de Informação em Saúde para a Atenção Básica* – Sisab)^14^. Finally, information on enrollment coverage in basic education was obtained from the school census conducted by the National Institute for Educational Studies and Research Anísio Teixeira (*Instituto Nacional de Estudos e Pesquisas Educacionais Anísio Teixeira* – INEP), under the Ministry of Education of Brazil^15^; while population data for the 0- to 19-year age group were obtained from the Brazilian population census conducted by the Brazilian Institute of Geography and Statistics (*Instituto Brasileiro de Geografia e Estatística* – IBGE)^16^.

### Variables studied

For this study, the outcome was assessed using four variables:

1) Use of oral health services in the past year;
2) Access to oral health services in the past year;
3) Use of public oral health services in the past year;
4) Access to public oral health services in the past year.

Effective access to oral health services was defined as the use of a service after seeking care, that is, when an adolescent or young person reported seeking dental care and actually received care within the past year. This definition is consistent with Andersen’s Behavioral Model, in which access is operationalized through the use of health services, resulting from the interaction among factors that predispose individuals to seek care, conditions that facilitate or hinder access, and health needs that motivate such care-seeking behavior^2,3^.

The variables were independently defined based on Andersen’s Behavioral Model and organized into three theoretical dimensions: predisposing factors, enabling factors, and need factors. Predisposing factors included sociodemographic characteristics that may influence the propensity to use services, such as self-reported race/ethnicity (White, Brown, and Black). Enabling factors encompassed socioeconomic and contextual conditions that may expand or restrict access, including per capita household income (at or below the poverty line: ≤R$ 665; above the poverty line: >R$ 665), oral health coverage in Primary Care in the municipalities (≤70% and >70%), basic education enrollment coverage (≤85% and >85%), and type of municipality (capital or non-capital municipality). Finally, the need factor was represented by dental caries experience (caries-free or with caries experience), considered an objective indicator of the need for dental care. A description of the variables is provided in Table 1.

**Table 1:** Description and categorization of the variables used in the study.

Characteristic	Type	Description	Categories/Cutoff
Use of oral health services in the past year	Outcome	Self-reported seeking of dental care in the past year.	Yes/No
Access to oral health services in the past year	Outcome	Receipt of dental care after seeking care in the past year.	Yes/No
Use of public oral health services in the past year	Outcome	Seeking care within the public health system in the past year.	Yes/No
Access to public oral health services in the past year	Outcome	Receipt of care within the public health system after seeking care in the past year.	Yes/No
Dental caries experience	Independent (individual)	Presence or absence of dental caries experience.	Caries-free/With caries experience
Self-reported race/ethnicity	Independent (individual)	Self-reported classification of race/skin color.	White/Brown/Black
Per capita household income	Independent (individual)	Income category according to the poverty line.	≤R$665 / >R$665
Coverage of primary oral health care	Independent (contextual)	Proportion of the population covered by oral health teams.	≤70% / >70%
Basic education enrollment coverage (0–19 years)	Independent (contextual)	Proportion of the population enrolled in the basic education system.	≤85% / <85%
Municipality type	Independent (contextual)	Category of the municipality of residence.	State capital/Interior

#### Statistical analysis

The data were analyzed inferentially, and all variables were presented as weighted relative frequencies, given the complex sampling design of the SB Brasil 2023 survey.

In addition, binary logistic regression models were fitted to estimate the odds ratios (ORs) for the occurrence of two outcomes of interest, defined as follows:

1. Use of dental services in the past year.
2. Access to dental services in the past year.
3. Use of public dental services in the past year.
4. Access to public dental services in the past year.

The data were analyzed using IBM SPSS Statistics^®^ software, version 27 (IBM Corp., Armonk, NY, USA)^17^, using a significance level of 5% (p<0.05). The logistic regression results are presented by means of Odds Ratio accompanied by 95% confidence intervals (95%CI).

##### Data availability statement

the data that support the results of this study are publicly accessible and can be obtained from the official repositories of the databases used, without having any restrictions on their access.

## RESULTS

Table 2 presents the characteristics of the study sample (n=14,340) and the contextual variables analyzed. Most adolescents and young people had dental caries experience, belonged to families with a per capita household income above the poverty line, and self-identified as Brown. Regarding service utilization, seeking and accessing oral health services were less frequent in most cases. Among the contextual variables, municipalities with oral health coverage in Primary Care of up to 70%, higher enrollment coverage in basic education (>85%), and municipalities located in the interior predominated.

**Table 2:** Characteristics of the variables studied, with relative frequencies and confidence intervals, according to data from the SB Brasil 2023 survey, for the adolescent and young adult population.

Individual characteristics	Relative frequency (CI)
Dental caries experience	
Caries-free	36.4 (36.4; 36.4)
With caries experience	63.6 (63.6; 63.6)
Per capita household income	
At or below the poverty line	40.1 (40.1; 40.2)
Above the poverty line	59.9 (59.8; 59.9)
Self-reported race/ethnicity	
White	39.0 (38.9; 39.0)
Black	12.4 (12.3; 12.4)
Brown	48.7 (48.6; 48.7)
Sought oral health services in the past year	
Yes	61.4 (61.4; 61.5)
No	38.6 (38.5; 38.6)
Accessed oral health services in the past year	
Yes	53.6 (53.6; 53.7)
No	46.4 (46.3; 46.4)
Sought public oral health services in the past year	
Yes	47.9 (47.8; 47.9)
No	52.1 (52.1; 52.2)
Accessed public oral health services in the past year	
Yes	43.7 (43.7; 43.8)
No	56.3 (56.2; 56.3)
Contextual characteristics	
Primary oral health care coverage	
Up to 70%	59.9 (59.8; 59.9)
>70%	40.1 (40.1; 40.2)
Basic education enrollment coverage	
Up to 85%	42.7 (42.7; 42.7)
>85%	57.3 (57.3; 57.3)
City type	
State capital	21.3 (21.3; 21.3)
Interior	78.7 (78.7; 78.7)

CI: Confidence interval.

The adjusted logistic regression models identified statistically significant associations between all variables analyzed. Regarding the seeking of oral health services among adolescents and young people, the likelihood of seeking care was higher among those with dental caries experience (OR=1.527) and those residing in municipalities in the interior (OR=1.544). In contrast, respondents with Black race/skin color (OR=0.864) and those with a per capita household income below the poverty line (OR=0.843) were associated with lower odds of seeking oral health services (Table 3).

**Table 3:** Logistic regression analysis of factors associated with seeking oral health services in the past year among adolescents and young adults in Brazil.

	Odds Ratio	95%CI		p-value
		Lower	Upper	
Dental caries experience (Caries-free)	1			
With caries experience	1.527	1.524	1.531	<0.001
Per capita household income (Above the poverty line)	1			
Below the poverty line	0.843	0.841	0.845	<0.001
Self-reported race/ethnicity (White)	1			
Black	0.864	0.861	0.867	<0.001
Brown	0.992	0.990	0.994	<0.001
Oral health coverage (>70%)	1			
<70%	1.044	1.041	1.046	<0.001
Basic education enrollment coverage (>85%)	1			
<85%	1.102	1.099	1.104	<0.001
City type (State capital)	1			
Interior	1.544	1.540	1.548	<0.001

95%CI: 95% Confidence interval.

Table 4 presents the results of the logistic regression analysis of factors associated with effective access to oral health services among adolescents and young people in Brazil. Although the variables show the same direction of association observed for seeking care (Table 3), differences in the magnitude of the effects can be observed. Access appears to widen racial disparities: the barrier faced by respondents with Black race/skin color became more pronounced, with access being more difficult (OR=0.789) than seeking care (OR=0.864). Factors such as dental caries experience (OR=1.434) and residing in the interior (OR=1.445) continue to be important facilitators of access, although with a slightly weaker strength of association compared with the seeking stage. These findings suggest that the access stage acts as a filter that deepens socioracial inequities.

**Table 4:** Logistic regression analysis of factors associated with access to oral health services in the past year among adolescents and young adults in Brazil.

	Odds Ratio	95%CI		p-value
		Lower	Upper	
Dental caries experience (Caries-free)	1			
With caries experience	1.434	1.431	1.437	<0.001
Per capita household income (Above the poverty line)	1			
Below the poverty line	0.863	0.862	0.865	<0.001
Self-reported race/ethnicity (White)	1			
Black	0.789	0.787	0.792	<0.001
Brown	0.948	0.946	0.950	<0.001
Oral health coverage (>70%)	1			
<70%	1.085	1.083	1.088	<0.001
Basic education enrollment coverage (>85%)	1			
<85%	1.058	1.056	1.061	<0.001
City type (State capital)	1			
Interior	1.445	1.442	1.449	<0.001

95%CI: 95% confidence interval.

Table 5 presents the results of the logistic regression analysis of factors specifically associated with seeking public oral health services among adolescents and young people. The model demonstrated a significant shift in the role of socioeconomic variables compared with the seeking of general oral health services (Table 3). The Black racial category emerged as the factor with the greatest magnitude of association with seeking public services (OR=2.720), as did per capita household income below the poverty line (OR=2.297), in contrast to the negative effect observed for general service-seeking. Supply-related factors, such as low oral health coverage in Primary Care (OR=0.972) and residing in the interior (OR=0.829), were associated with lower service-seeking. These results confirm that the public oral health network is the main resource sought by the most vulnerable population groups.

**Table 5:** Logistic regression analysis of factors associated with seeking public oral health services in the past year among adolescents and young adults in Brazil.

	Odds Ratio	95%CI		p-value
		Lower	Upper	
Dental caries experience (Caries-free)	1			
With caries experience	1.790	1.785	1.795	<0.001
Per capita household income (Above the poverty line)	1			
Below the poverty line	2.313	2.307	2.319	<0.001
Self-reported race/ethnicity (White)	1			
Black	2.717	2.705	2.729	<0.001
Brown	1.632	1.627	1.637	<0.001
Oral health coverage (>70%)	1			
<70%	0.508	0.507	0.510	<0.001
Basic education enrollment coverage (>85%)	1			
<85%	0.972	0.969	0.975	<0.001
City type (State capital)	1			
Interior	0.829	0.826	0.832	<0.001

95%CI: 95% confidence interval.

Table 6 presents the results of the logistic regression analysis of factors associated with effective access to public oral health services, allowing comparison with the results of the seeking stage (Table 5). The access model maintains the same direction of association for all variables, with the most vulnerable groups demonstrating a greater likelihood of using the service. However, access appears to act as a filter that modulates the intensity of the observed inequities. While the effect of low income is slightly more pronounced for access (OR=2.375 vs. OR=2.313 for seeking), the racial disparity among respondents with Black skin color is attenuated (the OR for access is 2.567, lower than the OR for seeking of 2.717). Similarly, the geographic barrier for those residing in the interior and the influence of low oral health coverage in Primary Care become stronger at the access stage. These results suggest that, after the intention to seek the service is manifested, the public health system is able to reduce racial inequality to some extent, but not socioeconomic or geographic inequality.

**Table 6:** Logistic regression analysis of factors associated with access to public oral health services in the past year among adolescents and young adults in Brazil.

	Odds Ratio	95%CI		p-value
		Lower	Upper	
Dental caries experience (Caries-free)	1			
With caries experience	1.799	1.793	1.805	<0.001
Per capita household income (Above the poverty line)	1			
Below the poverty line	2.375	2.368	2.382	<0.001
Self-reported race/ethnicity (White)	1			
Black	2.567	2.554	2.579	<0.001
Brown	1.563	1.559	1.568	<0.001
Oral health coverage (>70%)	1			
<70%	0.479	0.478	0.481	<0.001
Basic education enrollment coverage (>85%)	1			
<85%	0.880	0.878	0.883	<0.001
City type (State capital)	1			
Interior	0.785	0.782	0.788	<0.001

95%CI: 95% confidence interval.

## DISCUSSION

The results of this study indicate that fewer than two-thirds of the population seek oral health services, revealing a discrepancy between the need for care and its effective utilization. This trend reinforces the persistence of inequalities in access to oral health services among young Brazilians, a phenomenon documented in different contexts within the SUS^6,7^. This may contribute, as suggested by the data from this study, to the high prevalence of dental caries among adolescents and young people.

Notably, racial and socioeconomic inequalities continue to shape the main gaps in exclusion: being poor or having an income below the poverty line significantly reduces the likelihood of seeking dental services, confirming evidence that structural and cultural barriers limit access among socially vulnerable and low-income populations^18,19^.

These findings can be understood in light of the Modified Andersen Behavioral Model, according to which the use of health services results from the interaction among factors that predispose individuals to seek care, conditions that facilitate or hinder access, and health needs that motivate care-seeking behavior^2,3^. In the present study, race/skin color and income acted as barriers from the initial stage of seeking care, whereas the presence of dental caries functioned as a need that drives care-seeking. Furthermore, the greater use of public services by the most vulnerable groups highlights the role of the SUS in reducing disparities at the access stage, while also underscoring the persistence of socioeconomic and territorial barriers that must be addressed to consolidate equity in oral health.

An analysis of the contextual variables shows that low coverage of the Family Health Strategy (FHS) by Oral Health Teams (OHTs) in large urban centers constitutes a relevant determinant of the observed inequalities. Research shows that the concentration of the FHS in peripheral areas and the shortage of health facilities in metropolitan areas result in fragmented Primary Health Care (PHC), with difficulties in consolidating the territorialization of care and ensuring continuous follow-up of adolescents and young people^20^. These findings reinforce the urgency of public policies capable of addressing underfunding, reducing staff turnover, and adapting the FHS model to the heterogeneity and complexity of urban dynamics^21,22^.

The persistence of racial inequalities at the access stage, with greater barriers among those who identify as Black, suggests that the health system still operates as a social filter, reproducing historical and racial hierarchies that are also manifested in therapeutic care pathways^23^. The fact that lower-income and self-identified low-income individuals are more likely to use the public sector reinforces the role of SUS as the main mechanism for mitigating inequities, even though it remains constrained by territorial inequalities in the availability and distribution of services^24,25^.

These findings are discussed with reference to the concept of equity in health, understood as an ethical and political commitment to reducing inequalities among population groups^26,27^. In the Brazilian context, the principle of equity is embedded in the structure of PHC and guides the prioritization of the most vulnerable populations. However, as Buss and Pellegrini Filho point out, the implementation of this approach depends on the capacity for intersectoral coordination, public financing, and care models that recognize and incorporate the social, territorial, and cultural diversity of the country^28^.

Regarding the limitations, potential selection biases that may affect the representation of more remote areas should be considered, as well as measurement biases, given that the data were self-reported and involved multiple respondents. It is also important to consider that traditional logistic regression, which is suitable for estimating associations between independent variables and binary outcomes, may not fully capture hierarchical structures or broader contextual effects, particularly when individuals are nested within different municipal contexts. Thus, the absence of multilevel modeling may limit the understanding of contextual variability, representing a methodological limitation to be considered in future studies.

The methodological advances incorporated into the most recent national surveys, such as SB Brasil 2023, represent significant gains for oral health surveillance in the country, enhancing the precision of estimates and allowing greater temporal comparability. Because it is a nationally representative survey conducted with methodological rigor, its findings have high external validity and can inform public policies aimed at promoting equity in oral health^11,12^.

The findings of this study reinforce the persistence of racial, socioeconomic, and territorial inequalities in seeking and accessing oral health services among adolescents and young people in Brazil, with the consequent persistence of the disease burden. Despite the compensatory role of SUS, there is an urgent need to expand the qualified provision of services, with a focus on reducing structural barriers and advancing equity in oral health care.
