# Supplementary material for: Factors associated with seeking and access to oral health services among Brazilian adolescents and young adults
Source: Rev Bras Epidemiol. 2026 Aug 28;29(Suppl 1):e260032supl1. doi: 10.1590/1980-549720260032.supl.1 (PMC13523784; doi:10.1590/1980-549720260032.supl.1)
Supplement: Translation

# Translation: Fatores associados à procura e ao acesso aos serviços de saúde bucal entre adolescentes e jovens brasileiros

**DOI:** 10.1590/1980-549720260032.supl.1.2

**Authors:** Rênnis Oliveira da Silva, Carolina Thaiza Costa Pazos, Ana Beatriz Vasconcelos Lima Araújo, Nilcema Figueiredo, Gabriela da Silveira Gaspar, Paulo Sávio Angeiras de Goes

**Affiliations:** I Universidade Federal de Pernambuco, Programa de Pós-Graduação em Saúde da Criança e do Adolescente – Recife (PE), Brasil.; II Universidade Federal de Pernambuco, Departamento de Medicina Social – Recife (PE), Brasil.; III Universidade Federal de Pernambuco, Departamento de Saúde Coletiva, Centro Acadêmico de Vitória – Vitória de Santo Antão (PE), Brasil.; IV Universidade Federal de Pernambuco, Departamento de Clínica e Odontologia Social – Recife (PE), Brasil.

**Keywords:** Equidade no acesso aos serviços de saúde, Saúde bucal, Inquéritos de saúde bucal, Adolescentes

## Abstract

**Objetivo:**

Investigar os fatores individuais e contextuais associados à procura e ao acesso aos serviços de saúde bucal entre adolescentes e jovens brasileiros.

**Métodos:**

Estudo transversal realizado com base na Pesquisa Nacional de Saúde Bucal (SB Brasil) 2023. A amostra foi composta por adolescentes de 12 anos e jovens de 15 a 19 anos (n=14.757). Avaliaram-se quatro desfechos estruturados em duas etapas (procura e acesso), subdivididos em serviços de saúde bucal gerais e especificamente públicos. Foram ajustados modelos de regressão logística binária, estimando-se a razão de chances (*Odds Ratio* – OR), e adotado o nível de significância de 5% (p<0,05).

**Resultados:**

Todas as associações foram estatisticamente significativas (p<0,001). A cor preta (OR=0,86) e a renda abaixo da linha da pobreza (OR=0,84) reduziram a chance de procura geral por serviços de saúde bucal. No acesso geral, as iniquidades sociorraciais se aprofundaram, intensificando a barreira para o grupo preto (OR=0,79). Inversamente, no contexto dos serviços públicos, os grupos mais vulnerabilizados apresentaram as maiores chances tanto de procura quanto de acesso efetivo, destacando-se a cor preta (OR_procura_ = 2,72; OR_acesso_ = 2,57) e a baixa renda (OR_procura_ = 2,31; OR_acesso_ = 2,38).

**Conclusões:**

A procura e o acesso aos serviços de saúde bucal são influenciados por iniquidades socioeconômicas e raciais. O sistema público atua como um recurso compensatório para grupos vulnerabilizados, mas ainda não é suficiente para superar as barreiras de acesso.

## INTRODUÇÃO

A saúde bucal é um componente essencial da saúde geral e do bem-estar físico, mental e social. Envolve a capacidade de falar, sorrir, mastigar, saborear, tocar, engolir e expressar emoções com confiança, sem dor, desconforto ou doenças no complexo craniofacial^1^. O acesso aos serviços de saúde tem sido demonstrado como um fenômeno complexo, o qual tem sido analisado sob várias perspectivas. Sua compreensão foi sistematizada pelo Modelo Comportamental de Andersen^2^ e Andersen Modificado^3^, amplamente utilizado para explicar os determinantes do uso dos serviços de saúde. Esse modelo teórico também tem sido aplicado na análise do acesso aos cuidados em saúde bucal, permitindo explicar como fatores predisponentes, facilitadores e necessidades influenciam o uso dos serviços odontológicos^4,5^.

A adolescência e a juventude constituem fases críticas do curso da vida, marcadas por intensas transformações comportamentais, aquisição progressiva de autonomia e consolidação de hábitos de saúde. A forma como o cuidado odontológico ocorre nesse período pode repercutir ao longo da vida adulta, uma vez que necessidades não atendidas tendem a se agravar e ampliar desigualdades em saúde bucal^6,7^.

No Brasil, o cuidado em saúde bucal é garantido pelo Sistema Único de Saúde (SUS), por meio da Política Nacional de Saúde Bucal, instituída em 2004 e que se tornou a Lei n.º 14.572, de 8 de maio de 2023, conhecida como Brasil Sorridente^8,9^. Apesar disso, o acesso aos serviços de saúde bucal ainda apresenta desigualdades importantes, especialmente entre grupos com baixa renda, escolaridade reduzida e residentes em territórios vulnerabilizados^6,8^.

Considerando a persistência das desigualdades e a escassez de investigações que avaliem simultaneamente procura e acesso sob a perspectiva de determinantes individuais e contextuais, este estudo parte da hipótese de que vulnerabilidades socioeconômicas e raciais reduzem a probabilidade de utilização dos serviços, enquanto fatores estruturais, como cobertura assistencial e inserção escolar, podem atuar como importantes mediadores de equidade. Aliado a isso, a busca da construção de modelos de organização da saúde bucal baseados em componentes que promovam equidade tem se colocado como imperativo para a pesquisa em saúde bucal na América Latina^10^.

Diante do exposto, o objetivo deste estudo foi investigar os fatores individuais e contextuais associados à procura e ao acesso aos serviços de saúde bucal entre adolescentes e jovens brasileiros.

## MÉTODOS

### Delineamento do estudo

Estudo observacional, transversal, de natureza descritiva e analítica, realizado com base nos microdados da Pesquisa Nacional de Saúde Bucal (SB Brasil) 2023^11^. Informações detalhadas sobre os aspectos metodológicos e o desenho amostral do inquérito estão disponíveis nas publicações de Vargas et al.^12^ e Alves et al^13^.

O SB Brasil 2023 seguiu os princípios éticos estabelecidos na Resolução n.º 466/2012 do Conselho Nacional de Saúde, tendo sido aprovado pelo Comitê Nacional de Ética em Pesquisa (Parecer n.º 4.823.054). A participação dos respondentes foi voluntária, mediante assinatura do Termo de Consentimento Livre e Esclarecido (TCLE) e do Termo de Assentimento Livre e Esclarecido (TALE), quando aplicável.

### Amostra

A amostra do presente estudo foi composta por adolescentes de 12 anos e jovens de 15 a 19 anos que responderam ao questionário individual do SB Brasil 2023 (n=14.757). A amostra do inquérito foi selecionada por meio de amostragem probabilística estratificada por conglomerados (setores censitários), garantindo representatividade populacional^12,13^. O plano amostral do SB Brasil 2023 contemplou 53 domínios geográficos, correspondentes aos 26 estados, ao Distrito Federal e às capitais estaduais^13^.

### Coleta de dados

Os dados individuais (procura e acesso aos serviços de saúde bucal, experiência de cárie, renda domiciliar per capita e declaração étnico-racial) e contextuais (tipo de município) foram coletados do banco de dados da Pesquisa Nacional de Saúde Bucal^14^.

As demais informações contextuais sobre a cobertura de saúde bucal da Atenção Primária foram obtidas por meio do Sistema de Informação em Saúde para a Atenção Básica (Sisab)^14^. Por fim, as informações sobre a cobertura de matrículas na educação básica foram obtidas no censo escolar do Instituto Nacional de Estudos e Pesquisas Educacionais Anísio Teixeira (INEP), do Ministério da Educação do Brasil^15^; e a população na faixa etária de 0 a 19 anos, no censo da população brasileira do Instituto Brasileiro de Geografia e Estatística (IBGE)^16^.

### Variáveis estudadas

Para este estudo, avaliou-se o desfecho com quatro variáveis:

1) Procura aos serviços de saúde bucal no último ano;
2) Acesso aos serviços de saúde bucal no último ano;
3) Procura aos serviços públicos de saúde bucal no último ano;
4) Acesso aos serviços públicos de saúde bucal no último ano.

O acesso efetivo aos serviços de saúde bucal foi definido como a utilização do serviço após a procura, ou seja, quando o adolescente ou jovem relatou ter buscado atendimento odontológico e efetivamente foi atendido no último ano. Essa definição está alinhada ao Modelo Comportamental de Andersen, no qual o acesso se concretiza na etapa de uso dos serviços de saúde, sendo resultado da interação entre fatores que predispõem o indivíduo ao cuidado, condições que facilitam ou dificultam o acesso e necessidades de saúde que motivam a busca^2,3^.

As variáveis independentes foram definidas com base no Modelo Comportamental de Andersen, sendo organizadas em três dimensões teóricas: fatores predisponentes, fatores facilitadores e fatores de necessidade. Como fatores predisponentes, foram incluídas características sociodemográficas que podem influenciar a propensão ao uso de serviços, como declaração étnico-racial (brancos, pardos e pretos). Os fatores facilitadores contemplaram condições socioeconômicas e contextuais que podem ampliar ou restringir o acesso, incluindo renda domiciliar per capita (até a linha da pobreza: ≤R$ 665; acima da linha da pobreza: >R$ 665), cobertura da Atenção Básica em Saúde Bucal nos municípios (≤70% e >70%), cobertura de matrícula na educação básica (≤≤85% e >85%) e tipo de município (capital ou interior). Por fim, o fator de necessidade foi representado pela experiência de cárie (livre de cárie ou com experiência de cárie), considerada um indicador objetivo da necessidade de cuidado odontológico. A descrição das variáveis está presente na Tabela 1.

**Tabela 1:** Descrição e categorização das variáveis utilizadas no estudo.

Variável	Tipo	Descrição	Categorias/Corte
Procura por serviços de saúde bucal no último ano	Desfecho	Relato de busca por atendimento odontológico no último ano.	Sim/Não
Acesso aos serviços de saúde bucal no último ano	Desfecho	Realização de atendimento odontológico após a procura no último ano.	Sim/Não
Procura por serviços públicos de saúde bucal no último ano	Desfecho	Busca por atendimento na rede pública de saúde no último ano.	Sim/Não
Acesso aos serviços públicos de saúde bucal no último ano	Desfecho	Atendimento realizado na rede pública após a procura no último ano.	Sim/Não
Experiência de cárie	Independente (individual)	Presença ou ausência de experiência de cárie.	Livre de cárie/Com experiência de cárie
Autodeclaração étnico-racial	Independente (individual)	Classificação autorreferida de raça/cor.	Branca/Parda/Preta
Renda domiciliar per capita	Independente (individual)	Faixa de renda segundo linha de pobreza.	≤R$665 / >R$665
Cobertura da atenção básica em saúde bucal	Independente (contextual)	Proporção populacional coberta por equipes de saúde bucal.	≤70% / >70%
Cobertura de matrícula na educação básica (0-19 anos)	Independente (contextual)	Proporção populacional matriculada na rede de educação básica.	≤85% / <85%
Tipo de município	Independente (contextual)	Categoria do município de residência.	Capital/Interior

#### Análises estatísticas

Os dados foram analisados de forma inferencial e optou-se por apresentar todas as variáveis em frequências relativas ponderadas, dado o arranjo de amostra complexa do SB Brasil 2023.

Além disso, foram ajustados modelos de regressão logística binária, estimando-se a razão de chances (*Odds Ratio* – OR) para a ocorrência dos desfechos de interesse, definidos como:

1. Procura por serviços odontológicos no último ano.
2. Acesso aos serviços odontológicos no último ano.
3. Procura pelo serviço odontológico público no último ano.
4. Acesso aos serviços odontológicos públicos no último ano.

Os dados foram analisados no software *IBM SPSS Statistics* ^®^, versão 27 (IBM Corp., Armonk, NY, EUA)^17^, utilizando um nível de significância de 5% (p<0,05). Os resultados da regressão logística foram expostos por meio de *Odds Ratio* acompanhados com intervalos de confiança de 95% (IC95%).

##### Declaração de disponibilidade de dados

os dados que sustentam os resultados deste estudo são de acesso público e podem ser obtidos nos repositórios oficiais das bases de dados utilizadas, não havendo restrições ao seu acesso.

## RESULTADOS

A Tabela 2 apresenta as características da amostra do estudo (n=14.340) e das variáveis contextuais analisadas. Observa-se predominância de adolescentes e jovens com experiência de cárie, pertencentes a famílias com renda domiciliar per capita acima do nível de pobreza e autodeclarados pardos. Em relação à utilização dos serviços, a maioria relatou ter procurado e acessado serviços de saúde bucal, embora a busca e o acesso aos serviços públicos tenham sido menos frequentes. Para as variáveis contextuais, prevaleceram localidades com cobertura de saúde bucal na atenção básica de até 70%, maior cobertura de matrícula na educação básica acima de 85% e municípios situados no interior.

**Tabela 2:** Características das variáveis estudadas com frequências relativas e intervalo de confiança, segundo os dados do SB Brasil 2023, para a população de adolescentes e jovens.

Variáveis individuais	Frequência relativa (IC)
Experiência de cárie	
Livre de cárie	36.4 (36.4; 36.4)
Com experiência de cárie	63.6 (63.6; 63.6)
Renda domiciliar per capita	
Até o nível de pobreza	40.1 (40.1; 40.2)
Acima do nível da pobreza	59.9 (59.8; 59.9)
Declaração étnico-racial	
Brancos	39.0 (38.9; 39.0)
Pretos	12.4 (12.3; 12.4)
Pardos	48.7 (48.6; 48.7)
Procurou serviço de saúde bucal no último ano	
Sim	61.4 (61.4; 61.5)
Não	38.6 (38.5; 38.6)
Acessou serviço de saúde bucal no último ano	
Sim	53.6 (53.6; 53.7)
Não	46.4 (46.3; 46.4)
Procurou serviço público de saúde bucal no último ano	
Sim	47.9 (47.8; 47.9)
Não	52.1 (52.1; 52.2)
Acessou serviço público de saúde bucal no último ano	
Sim	43.7 (43.7; 43.8)
Não	56.3 (56.2; 56.3)
Variáveis contextuais	
Cobertura saúde bucal AB	
Até 70%	59.9 (59.8; 59.9)
>70%	40.1 (40.1; 40.2)
Cobertura de matrícula na educação básica	
Até 85%	42.7 (42.7; 42.7)
>85%	57.3 (57.3; 57.3)
Tipo da cidade	
Capital	21.3 (21.3; 21.3)
Interior	78.7 (78.7; 78.7)

IC: Intervalo de confiança; AB: Atenção Básica.

As regressões logísticas ajustadas identificaram associações estatisticamente significativas entre todas as variáveis analisadas. Quanto à procura por serviço de saúde bucal entre adolescentes e jovens, observou-se que aumenta a chance de procura: ter experiência de cárie (OR=1,527) e residir em município do interior (OR=1,544). Em contraste, respondentes com a cor de pele preta (OR=0,864) e com renda domiciliar per capita abaixo da linha da pobreza (OR=0,843) foram associados a uma menor chance de procurar o serviço (Tabela 3).

**Tabela 3:** Análise de regressão logística dos fatores associados à procura de serviço de saúde bucal, no último ano, por adolescentes e jovens no Brasil.

	*Odds Ratio*	IC95%		p-valor
		Inferior	Superior	
Experiência de cárie (Livre)	1			
Com experiência	1,527	1,524	1,531	<0,001
Renda domiciliar per capita (Acima da linha da pobreza)	1			
Abaixo da linha da pobreza	0,843	0,841	0,845	<0,001
Declaração étnico-racial (Branca)	1			
Preta	0,864	0,861	0,867	<0,001
Parda	0,992	0,990	0,994	<0,001
Cobertura de saúde bucal (>70%)	1			
<70%	1,044	1,041	1,046	<0,001
Cobertura de matrícula na educação básica (>85%)	1			
<85%	1,102	1,099	1,104	<0,001
Tipo de cidade (Capital)	1			
Interior	1,544	1,540	1,548	<0,001

IC95%: Intervalo de Confiança de 95%.

A Tabela 4 apresenta os resultados da regressão logística para os fatores associados ao acesso efetivo aos serviços de saúde bucal por adolescentes e jovens no Brasil. Embora as variáveis apresentem o mesmo sentido de associação observado na procura (Tabela 3), notam-se diferenças na magnitude dos efeitos. O acesso parece ampliar as disparidades raciais: a barreira para respondentes com cor de pele preta se acentuou, com o acesso sendo mais dificultado (OR=0,789) do que a procura (OR=0,864). Fatores como a experiência de cárie (OR=1,434) e residir no interior (OR=1,445) continuam sendo facilitadores importantes do acesso, porém com uma força de associação ligeiramente menor quando comparados à fase de procura. Estes achados sugerem que a etapa de acesso atua como um filtro que aprofunda as iniquidades sociorraciais.

**Tabela 4:** Análise de regressão logística dos fatores associados ao acesso aos serviços de saúde bucal, no último ano, por adolescentes e jovens no Brasil.

	*Odds Ratio*	IC95%		p-valor
		Inferior	Superior	
Experiência de cárie (Livre)	1			
Com experiência	1,434	1,431	1,437	<0,001
Renda domiciliar per capita (Acima da linha da pobreza)	1			
Abaixo da linha da pobreza	0,863	0,862	0,865	<0,001
Declaração étnico-racial (Branca)	1			
Preta	0,789	0,787	0,792	<0,001
Parda	0,948	0,946	0,950	<0,001
Cobertura de saúde bucal (>70%)	1			
<70%	1,085	1,083	1,088	<0,001
Cobertura de matrícula na educação básica (>85%)	1			
<85%	1,058	1,056	1,061	<0,001
Tipo de cidade (Capital)	1			
Interior	1,445	1,442	1,449	<0,001

IC95%: Intervalo de Confiança de 95%.

A Tabela 5 apresenta os resultados da regressão logística para os fatores associados especificamente à procura por serviços públicos de saúde bucal entre adolescentes e jovens. O modelo evidenciou uma inversão significativa no papel das variáveis socioeconômicas, em comparação com a procura por serviços em geral (Tabela 3). A categoria racial preta emerge como o fator de maior magnitude para a procura do serviço público (OR=2,720), assim como a renda domiciliar per capita abaixo da linha da pobreza (OR=2,297), diferentemente do efeito negativo observado na procura geral. Fatores relacionados à oferta, como baixa cobertura de saúde bucal na atenção básica (OR=0,972) e residir no interior (OR=0,829), associaram-se a uma menor procura. Estes resultados confirmam que a rede pública de saúde bucal é o principal recurso buscado pelos grupos populacionais mais vulnerabilizados.

**Tabela 5:** Análise de regressão logística dos fatores associados à procura por serviços públicos de saúde bucal, no último ano, por adolescentes e jovens no Brasil.

	*Odds Ratio*	IC95%		p-valor
		Inferior	Superior	
Experiência de cárie (Livre)	1			
Com experiência	1,790	1,785	1,795	<0,001
Renda domiciliar per capita (Acima da linha da pobreza)	1			
Abaixo da linha da pobreza	2,313	2,307	2,319	<0,001
Declaração étnico-racial (Branca)	1			
Preta	2,717	2,705	2,729	<0,001
Parda	1,632	1,627	1,637	<0,001
Cobertura de saúde bucal (>70%)	1			
<70%	0,508	0,507	0,510	<0,001
Cobertura de matrícula na educação básica (>85%)	1			
<85%	0,972	0,969	0,975	<0,001
Tipo de cidade (Capital)	1			
Interior	0,829	0,826	0,832	<0,001

IC95%: Intervalo de Confiança de 95%.

A Tabela 6 apresenta os resultados da regressão logística para o acesso efetivo aos serviços públicos de saúde bucal, permitindo a comparação com os resultados da fase de procura (Tabela 5). O modelo de acesso mantém a direção de associação para todas as variáveis, com os grupos mais vulneráveis demonstrando maior chance de utilizar o serviço. No entanto, o acesso parece atuar como um filtro que modula a intensidade das iniquidades observadas. Enquanto o efeito da baixa renda é ligeiramente mais acentuado no acesso (OR=2,375 vs. OR=2,313 na procura), a disparidade racial para os respondentes de cor preta é atenuada (OR de acesso é 2,567, menor que o OR de procura de 2,717). De forma semelhante, a barreira geográfica para o residente do interior e a influência da baixa cobertura de saúde bucal na atenção básica se tornam mais fortes na etapa de acesso. Estes resultados sugerem que, após a manifestação da intenção de buscar o serviço, o sistema de saúde público consegue reduzir, em certa medida, a desigualdade racial, mas não a socioeconômica e geográfica.

**Tabela 6:** Análise de regressão logística dos fatores associados ao acesso a serviços públicos de saúde bucal, no último ano, por adolescentes e jovens no Brasil.

	*Odds Ratio*	IC95%		p-valor
		Inferior	Superior	
Experiência de cárie (Livre)	1			
Com experiência	1,799	1,793	1,805	<0,001
Renda domiciliar per capita (Acima da linha da pobreza)	1			
Abaixo da linha da pobreza	2,375	2,368	2,382	<0,001
Declaração étnico-racial (Branca)	1			
Preta	2,567	2,554	2,579	<0,001
Parda	1,563	1,559	1,568	<0,001
Cobertura de saúde bucal (>70%)	1			
<70%	0,479	0,478	0,481	<0,001
Cobertura de matrícula na educação básica (>85%)	1			
<85%	0,880	0,878	0,883	<0,001
Tipo de cidade (Capital)	1			
Interior	0,785	0,782	0,788	<0,001

IC95%: Intervalo de Confiança de 95%.

## DISCUSSÃO

Os resultados deste estudo indicam que a procura pelos serviços de saúde bucal não atinge dois terços do público avaliado, revelando uma discrepância entre a necessidade e o uso efetivo do cuidado. Esse achado reforça a persistência de iniquidades no acesso aos serviços de saúde bucal entre jovens brasileiros, um fenômeno documentado em diferentes contextos do SUS^6,7^ – o que pode contribuir, inclusive, segundo os dados deste estudo, para uma alta prevalência de experiência de cárie entre adolescentes e jovens.

Notadamente, as desigualdades raciais e socioeconômicas continuam a configurar os principais eixos de exclusão: ser preto ou viver com renda abaixo da linha da pobreza reduziu significativamente a probabilidade de procurar o serviço odontológico, o que confirma evidências de que barreiras estruturais e culturais limitam o acesso de populações socialmente vulneráveis e de baixa renda^18,19^.

Esses achados podem ser compreendidos à luz do Modelo Comportamental de Andersen e Andersen Modificado, segundo os quais o uso dos serviços de saúde resulta da interação entre fatores que predispõem ao cuidado, condições que facilitam ou dificultam o acesso e necessidades de saúde que motivam a procura^2,3^. No presente estudo, raça/cor e renda atuaram como barreiras desde a etapa inicial de busca, enquanto a presença de cárie funcionou como necessidade que impulsiona o cuidado. Além disso, o maior uso do serviço público pelos grupos mais vulneráveis evidencia o papel do SUS na redução das disparidades na etapa de acesso, embora persistam obstáculos socioeconômicos e territoriais que precisam ser enfrentados para a consolidação da equidade em saúde bucal.

A análise das variáveis contextuais evidencia que a baixa cobertura da Estratégia Saúde da Família (ESF) com Equipes de Saúde Bucal (ESB) nos grandes centros urbanos constitui um determinante relevante das desigualdades observadas. Pesquisas mostram que a concentração da ESF em áreas periféricas e o déficit de equipes nas metrópoles produzem uma Atenção Primária à Saúde (APS) fragmentada, com dificuldades para consolidar a territorialização do cuidado e garantir o acompanhamento contínuo de adolescentes e jovens^20^. Esses achados reforçam a urgência de políticas públicas capazes de enfrentar o subfinanciamento, reduzir a rotatividade de profissionais e adaptar o modelo da ESF à heterogeneidade e à complexidade das dinâmicas urbanas^21,22^.

A permanência das desigualdades raciais na etapa de acesso, com maior restrição para aqueles que se declaram pretos, sugere que o sistema de saúde ainda opera como um filtro social, reproduzindo hierarquias históricas e raciais que também se manifestam nos itinerários terapêuticos^23^. O fato de que indivíduos com menor renda e autodeclarados pretos apresentam maior propensão ao uso do setor público confirma o papel do SUS como principal mecanismo de mitigação das iniquidades, ainda que limitado por desigualdades territoriais na oferta e distribuição dos serviços^24,25^.

Essas evidências dialogam com o referencial da equidade em saúde, compreendida como o compromisso ético e político de reduzir desigualdades entre grupos populacionais^26,27^. No contexto brasileiro, o princípio da equidade estrutura a atenção primária e orienta a priorização de populações mais vulnerabilizadas. Contudo, como observam Buss e Pellegrini Filho, a concretização dessa diretriz depende da capacidade de articulação intersetorial, de um financiamento estável e de modelos de atenção que reconheçam e incorporem a diversidade social, territorial e cultural do país^28^.

Quanto às limitações, é importante considerar possíveis vieses de seleção, que interfiram na representatividade de áreas mais remotas, e de aferição, visto que se dá por autorrelato e com múltiplos avaliadores. É importante considerar, também, que a regressão logística tradicional, embora adequada para estimar associações entre variáveis independentes e desfechos binários, pode não captar integralmente estruturas hierárquicas ou efeitos contextuais mais amplos, especialmente quando indivíduos estão inseridos em diferentes contextos municipais. Assim, a ausência de modelagem multinível pode limitar a compreensão da variabilidade contextual, representando uma limitação metodológica a ser considerada em estudos futuros.

Os avanços metodológicos incorporados nos inquéritos nacionais mais recentes, como o SB Brasil 2023, representam ganho significativo para a vigilância em saúde bucal no país, ao aprimorar a precisão das estimativas e permitir maior comparabilidade temporal. Por se tratar de uma amostra com representatividade nacional e rigor metodológico, os achados apresentam elevada validade externa e podem subsidiar políticas públicas direcionadas à equidade em saúde bucal^11,12^.

Os achados deste estudo reforçam a persistência de desigualdades raciais, socioeconômicas e territoriais na procura e no acesso à saúde bucal entre adolescentes e jovens no Brasil, com consequente manutenção do quadro de doenças. Apesar do papel compensatório do SUS, evidencia-se a necessidade urgente de ampliar a oferta qualificada de serviços, com foco na redução de barreiras estruturais e na efetivação da equidade no cuidado em saúde bucal.
